# The transcription factor Ahr1 governs the recruitment of neutrophils to the brain and clearance of *Candida albicans* during CNS infection

**DOI:** 10.1128/mbio.01492-26

**Published:** 2026-07-20

**Authors:** Rohan S. Wakade, Norma V. Solis, Laura C. Ristow, Melanie Wellington, Scott G. Filler, Damian J. Krysan

**Affiliations:** 1 Department of Pediatrics, Carver College of Medicine, University of Iowa 160412, Iowa City, Iowa, USA; 2 Division of Infectious Diseases, Lundquist Institute for Biomedical Innovation a Harbor-UCLA Medical Center 21640, Torrance, California, USA; 3 Department of Medicine, David Geffen School of Medicine at UCLA 12222, Los Angeles, California, USA; 4 Department of Molecular Physiology and Biophysics, Carver College of Medicine, University of Iowa 174490, Iowa City, Iowa, USA; Instituto Carlos Chagas, Curitiba, Brazil

**Keywords:** *Candida albicans*, fungal pathogenesis, inflammasome, fungal brain infection

## Abstract

*Candida albicans* causes infections of the central nervous system in premature infants and people with primary immune deficiencies affecting the innate immune system. Compared to infections in other organs, the pathogenesis and host responses to *C. albicans* brain infections are understudied. However, the CARD9/NLRP3/interleukin 1 (IL-1) axis has been shown to play a central role in protecting the brain from *C. albicans* through the recruitment of neutrophils. In addition, Ece1/candidalysin is a key fungal trigger of this pathway in the brain. Here, we demonstrate that the transcription factor Ahr1 is also required for the activation of the NLRP3/IL-1β axis, recruitment of neutrophils, and clearance of *C. albicans* from the brain. In contrast to its function *in vitro*, Ahr1 does not govern the expression of *ECE1* in infected brain tissue. While our data show that Ahr1 and Ece1 have overlapping effects on the NLRP3/IL-1 axis, we have also observed clear distinctions in their function with respect to chemokine secretion. Our data support a model in which Ahr1 is required for the first priming step of NLRP3 inflammasome assembly, leading to IL-1β production. Ece1/candidalysin, on the other hand, plays an important role in driving both the second step of NLRP3 inflammasome activation and the inflammasome-independent IL-1α-mediated responses to *C. albicans* brain infection.

**IMPORTANCE:**

*Candida albicans* infections of the brain can cause devastating consequences, particularly for premature infants. However, *C. albicans* infection of the brain has been understudied relative to other types of infection. We have found that the regulator of gene expression Ahr1 governs the expression of *C. albicans* factors that are recognized by the host and which drive the influx of neutrophils to the brain. This process is critical to the clearance of the fungus from the brain. Our data also suggest that these Ahr1-dependent factors are required to prime this immune response and function at a different step compared to the toxin candidalysin, a protein that is also required for the host to clear *C. albicans* from the brain.

## INTRODUCTION

*Candida albicans* is one of the most common human fungal pathogens (1, 2). Disease-causing *C. albicans* infections involve a range of organ systems and affect people with multiple predisposing risk factors. In immunocompetent people, the majority of *C. albicans* infections involve mucosal surfaces or skin and include diaper dermatitis, thrush, and vulvovaginal candidiasis (3). Patients without specific alterations to immune function can also develop invasive, life-threatening infections (4). In these cases, the most common risk factors are recent abdominal surgery or the presence of an indwelling vascular catheter. The setting of altered immune function leads to two general types of candidiasis. Patients with altered T-cell functions, including, for example, people living with HIV/AIDS, have increased rates of oropharyngeal and esophageal candidiasis but do not typically develop invasive disease (5). In contrast, reduced innate immune function, most commonly due to neutropenia induced by cytotoxic chemotherapy, leads to increased rates of *C. albicans* bloodstream and deep organ infection (1, 4).

In very specific patient populations, *C. albicans* can cause central nervous system (CNS) infections (6–8). For example, it has long been recognized that premature infants have very high rates of CNS candidiasis compared to almost any other patient population (6, 8). CNS candidiasis almost never develops in older patients, even in the setting of prolonged neutropenia or chronic immunosuppression. In contrast, *C. albicans* is the second most common cause of late-onset meningitis in premature infants (8). Unfortunately, the outcomes of *C. albicans* CNS infection in premature infants are very poor, with high rates of mortality and neurodevelopmental deficits in survivors (8). In recent years, an additional group of patients with mutations in the CARD9-IL1 innate immune axis has been found to have an increased incidence of CNS candidiasis (7).

Compared to *C. albicans* infections of other organs, much less is known about the host and pathogen factors that contribute to CNS candidiasis. Two seminal studies from the Lionakis lab have provided important insights into both the CNS immune responses to *C. albicans* as well as the fungal factors that trigger them (7, 9). In immunocompetent mice, *C. albicans* initially establishes infection of the brain parenchyma, but it is cleared by days 3–5 (10). Mice lacking components of the CARD9-Nlrp3-IL-1 innate immune response pathway do not clear the fungus (7, 9). This pathway leads to CXCL1 secretion, which, in turn, contributes substantially to the recruitment of neutrophils and clearance of the fungus. Drummond et al. also showed that the secreted *C. albicans* toxin candidalysin (*ECE1*) is an important trigger of the Nlpr3-IL-1-CXL1 component of the immune response, and *C. albicans* strains lacking the *ECE1* gene establish persistent brain infection with higher fungal burden (9).

As part of a project to identify *C. albicans* determinants of CNS infection, we examined the role of the transcription factor Ahr1. Previous work in multiple labs has shown that deletion of *AHR1* reduces the ability of *C. albicans* to trigger NLRP3-mediated IL-1β secretion and pyroptosis in macrophages (11, 12). In addition, Ruben et al. have reported *in vitro* data indicating that Ahr1 plays an important role in the regulation of *ECE1* expression (13). Here, we report that Ahr1 governs neutrophil recruitment to the brain in an IL-1 pathway-dependent manner but, surprisingly, does not contribute significantly to the regulation of *ECE1* expression during brain infection. We also provide evidence that Ahr1-dependent processes may be important for the priming step of NLRP3 inflammasome activation during *C. albicans* CNS infection.

## RESULTS

### Ahr1 is required for the clearance of *C. albicans* from the brain but does not affect fungal burden in the kidney

**Fig 1:**
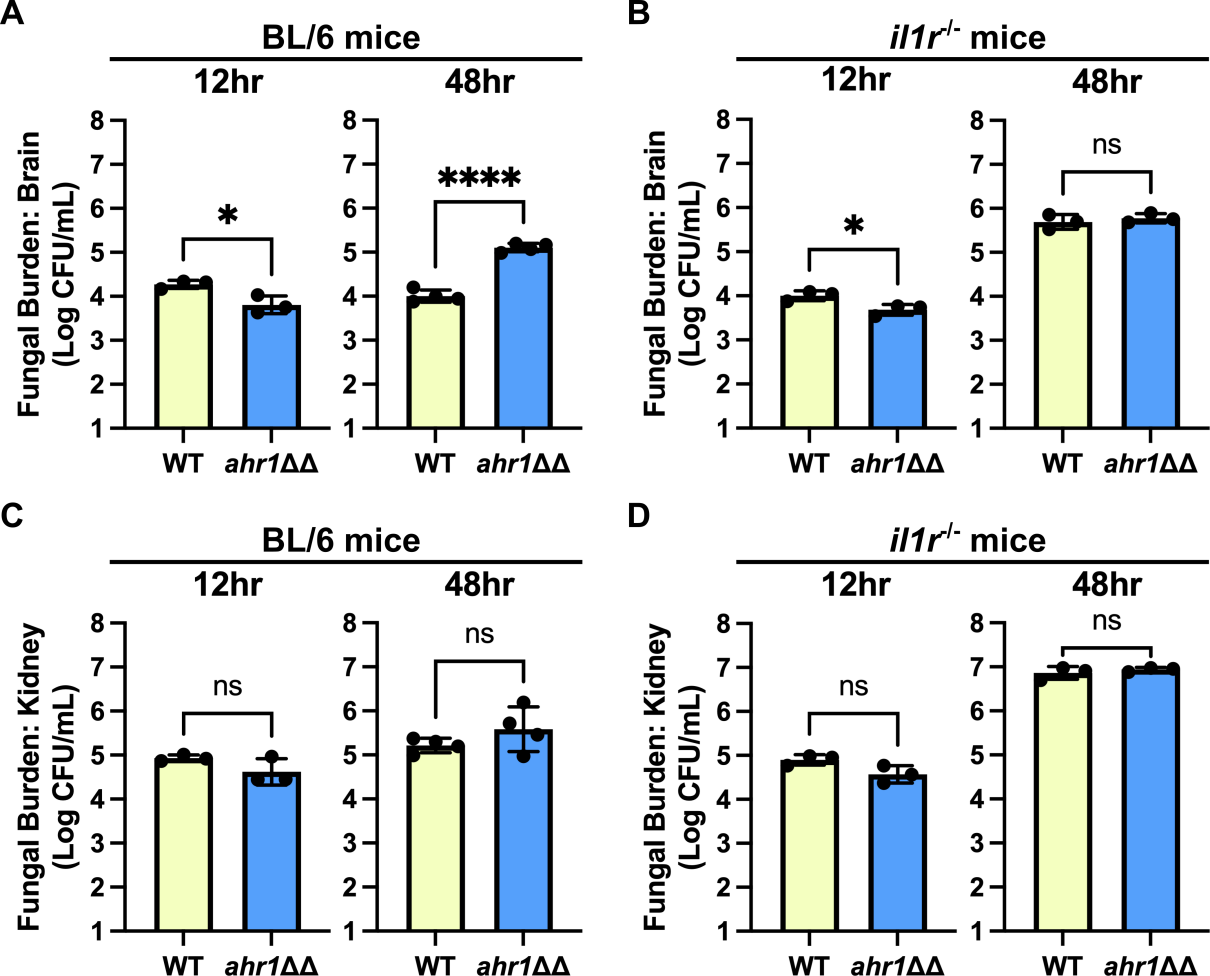
BL/6 but not *il1r^-^*^−/−^ mice infected with the *ahr1*∆∆ mutant have higher brain fungal burden than that of those infected with WT. BL/6 or *il1r*^−/−^ mice (*n* = 3–5/group) were infected with *C. albicans* WT or the *ahr1*∆∆ mutant by tail-vein injection, and brain (**A and C**) or kidney (**B and D**) fungal burden was determined at 12 or 48 h post-infection. Statistical differences between groups were determined by two-tailed Student’s *t*-test on log-transformed fungal burden data (*P* < 0.05 defined as significant; *P < 0.05; **P < 0.01; ***P < 0.001; ****P < 0.0001).

We infected BL/6 mice with WT and *ahr1*∆∆ mutant strains generated in the SN genetic background by tail-vein injection, sacrificed the mice 12 and 48 h post-infection, and determined the fungal burden in the brains (Fig. 1A). Mice infected with the *ahr1*∆∆ mutant showed a modest but statistically significant reduction in fungal burden at 12 h. In contrast, the fungal burden of the *ahr1*∆∆ mutant was increased by ~1 log10 CFU/mL at 48 h, suggesting that the *ahr1*∆∆ mutant is either more fit in CNS tissue or is not cleared as efficiently, compared to WT. The increased fungal brain burden in mice infected with an *ahr1*∆∆ mutant at 48 h was confirmed with two additional isolates in the setting of a prototrophic *ahr1*∆∆ mutant and WT strains (Fig. S1). As discussed above, activation of the IL-1 cytokine axis is critical for the clearance of *C. albicans* from the brain, and Drummond et al. have shown that IL-1R is required for this process (9). If Ahr1 governs the expression of genes that activate the IL-1 system to drive CNS clearance, then the increased fungal burden observed in WT mice infected with the *ahr1*∆∆ mutant should be reduced in mice that lack IL-1R.

To test this hypothesis, we infected *il1r*^−/−^ mice with WT and the *ahr1*∆∆ mutant and determined fungal brain burden at 12 and 48 h (Fig. 1B). As observed for BL/6 mice, the 12 h fungal brain burden of *il1r*^−/−^ mice infected with the *ahr1*∆∆ mutant was reduced relative to those infected with WT. In contrast, the 48 h fungal brain burden for *il1r*^−/−^ mice with the *ahr1*∆∆ mutant was not statistically different from that of the WT. The IL1R dependence of the increased brain fungal burden observed in mice infected with the *ahr1*∆∆ mutant supports the conclusion that Ahr1 is required to trigger the clearance of *C. albicans*. Furthermore, this result is inconsistent with the *ahr1*∆∆ mutant having increased fitness in brain tissue because, if that were the case, then the phenotype would have been independent of IL1R.

Next, we asked if the effect of Ahr1 on *C. albicans* clearance was organ-specific by comparing the kidney fungal burdens using the same combinations of *C. albicans* and mouse strains. In BL/6 mice, deletion of *AHR1* did not change the fungal burden at either time point (Fig. 1C). Similarly, *il1r*^−/−^ mice infected with the *ahr1*∆∆ mutant had similar kidney fungal burden compared to those infected with the WT strain (Fig. 1D). This demonstrates that Ahr1 plays a brain-specific role in both the establishment of initial *C. albicans* infection and clearance of *C. albicans* from the brain.

### Ahr1 is required for neutrophil recruitment to the *C. albicans*-infected brain

**Fig 2:**
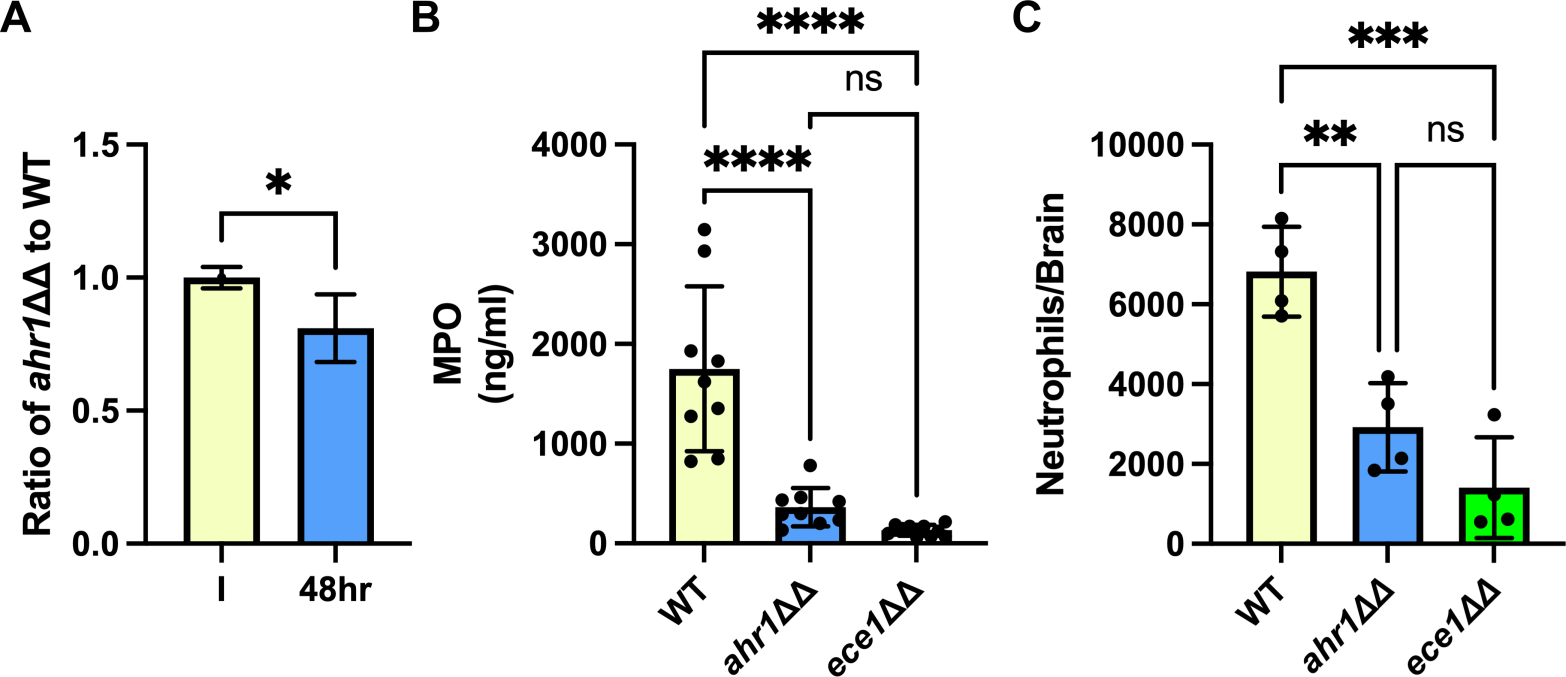
Ahr1 is required for neutrophil recruitment to *C. albicans*-infected brain tissue. (**A**) BL/6 mice (*n* = 4) were infected with a 1:1 mixture of WT and the *ahr1*∆∆ mutant by tail-vein injection, and brain tissue was harvested at 48 h post-infection. The ratio of ahr1ΔΔ mutant/WT was determined by RT-PCR using the actin gene for total fungal burden and the *ahr1*∆∆ barcode in the inoculum and normalized as 1 (noted as I in panel **A**). The ratio of the two strains was determined at 48 h using the same primers. (**B**) BL/6 mice (*n* = 10/group) were infected with the indicated strains by tail-vein injection, and brains were harvested 24 h post-infection. The tissue was homogenized and assayed for myeloperoxidase (MPO) activity as described in Materials and Methods. Differences between the groups were analyzed by one-way ANOVA and Tukey’s multiple comparisons test (****P = 0.0001). (**C**) BL/6 mice (two independent experiments with 2 mice/group) were infected with WT *C. albicans,* and brains were harvested 24 h post-infection. The number of neutrophils in the infected tissue was determined using a flow cytometry assay, as described in Materials and Methods. Differences between groups were analyzed by one-way ANOVA with Tukey’s multiple comparisons test (**P = 0.0028; ***P = 0.0003).

In principle, the reduced clearance of the *ahr1*∆∆ mutant could be due to reduced recruitment of neutrophils or resistance to neutrophil killing. To distinguish between these two possibilities, we carried out a competition experiment in which a 1:1 mixture of WT and a barcoded *ahr1*∆∆ mutant was used to inoculate BL/6 mice. *C. albicans* was isolated from the brain 48 h post-infection, and the ratio of WT to the *ahr1*∆∆ mutant was determined by quantitative PCR (Fig. 2A). The presence of the WT strain was sufficient to drive the clearance of the *ahr1*∆∆ mutant and abolish the 1 log10 CFU/mL increase in fungal burden observed for the *ahr1*∆∆ mutant relative to WT in the single-strain infections (Fig. 1A). If the *ahr1*∆∆ mutant was resistant to neutrophil-mediated killing, then the increase in fungal burden observed in the single strains would have persisted in the mixed-strain infection.

To further test the hypothesis that Ahr1 is required for neutrophil recruitment to *C. albicans*-infected brain tissue, we first compared the tissue levels of myeloperoxidase (MPO), an enzyme expressed by phagocytes, including neutrophils, in brain tissue infected with WT, the *ahr1*∆∆ mutant, and a strain lacking the gene (*ECE1*) encoding candidalysin (Ece1/CL). Consistent with our hypothesis, MPO concentrations were dramatically lower in the brains of mice infected with the *ahr1*∆∆ mutant compared to WT (Fig. 2B). Indeed, the MPO concentration in brain tissue infected with the *ahr1*∆∆ mutant was not statistically different from that of the *ece1*∆∆ mutant. To test our hypothesis more directly, we used flow cytometry to measure neutrophil recruitment to the brain tissue (gating strategy is shown in Fig. S2). In keeping with previously reported data (9), the brain tissue infected with the *ece1*∆∆ mutant contained far fewer neutrophils than tissue infected with the WT strain (Fig. 2C). Consistent with the MPO measurements, brain tissue infected with the *ahr1*∆∆ mutant also had reduced neutrophil counts relative to WT; the difference between the *ece1*∆∆ and *ahr1*∆∆ mutants did not reach statistical significance (Fig. 2C). Taken together, these data strongly support the conclusion that Ahr1 governs the expression of genes during brain infection that triggers IL1-mediated recruitment of neutrophils, leading to the clearance of *C. albicans* from the CNS.

### Ahr1 is required for *C. albicans* to induce IL1α/β secretion in brain tissue, but not CXCL1/2 or GM-CSF

**Fig 3:**
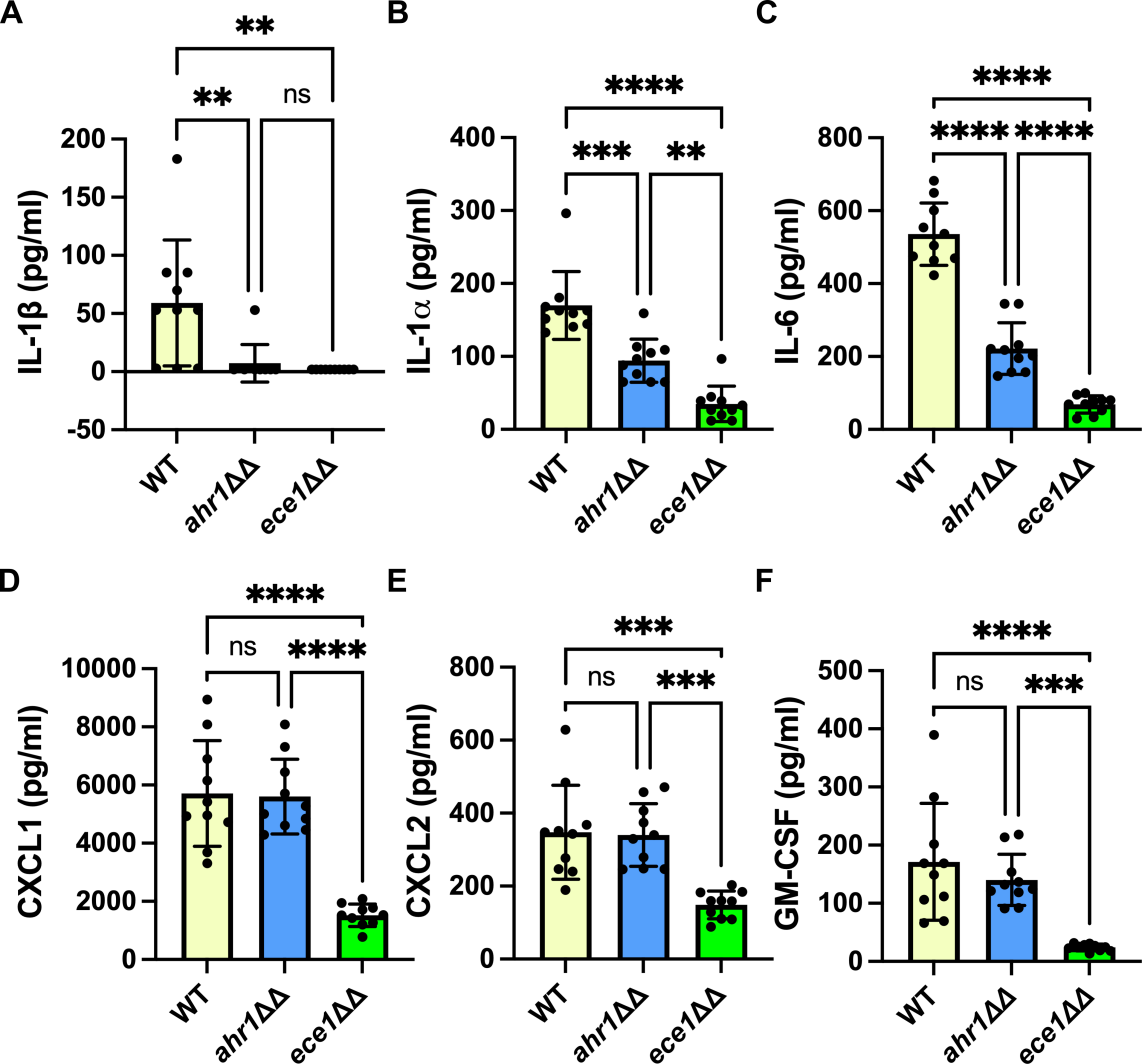
Ahr1 and Ece1 have both overlapping and distinct roles in triggering chemokine and cytokine production in *C. albicans*-infected brain tissue. (**A–F**) BL/6 mice (*n* = 10/group) were infected with the indicated strains by tail-vein injection, and brains were harvested 24 h post-infection. The tissue was homogenized and assayed for cytokine/chemokine concentration as described in Materials and Methods. Differences between groups were analyzed by one-way ANOVA and Tukey’s multiple comparisons test. (**A**) IL-1β concentrations: **P = 0.004 and 0.0016; NS, *P* > 0.05. (**B**) IL-1α concentrations: **P = 0.002; ***P = 0.001; ****P = 0.0001. (**C**) IL-6 ****P < 0.0001. (**D**) CXCL1 concentrations: ****P = 0.0001. (**E**) CXCL2 concentrations: ****P = 0.0002 and 0.0001. (**F**) GM-CSF concentrations: ***P = 0.001; ****P < 0.0001.

To further explore the mechanism by which Ahr1 affects neutrophil recruitment to the brain, we determined the levels of cytokines and chemokines involved in that process in mouse brains infected with WT, the *ahr1*∆∆ mutant, and the *ece1*∆∆ mutant. Brain tissue infected with both the *ahr1*∆∆ and *ece1*∆∆ mutants had very low amounts of the IL-1 family cytokine IL-1β compared to WT (Fig. 3A) (9). This is consistent with the observation that the differences in brain fungal burden between the WT and *ahr1*∆∆ mutant strains are eliminated in mice lacking the IL-1R. The levels of IL-1α were also reduced in the brain tissue infected with the mutants, but the *ece1*∆∆ mutant had a more pronounced effect than the *ahr1*∆∆ mutant (Fig. 3B). IL-6 levels were also reduced in the brain tissue of mice infected with the *ahr1*∆∆ mutant but not to the extent observed for mice infected with the *ece1*∆∆ mutant (Fig. 3C).

Drummond et al. reported that CXCL1 drives substantial neutrophil recruitment to the brain and that Ece1/CL is required for *C. albicans* to trigger its secretion (9). Consistent with their data, brain tissue infected with the *ece1*∆∆ mutant tissue showed significantly less CXCL1 than those infected with WT (Fig. 3D). Surprisingly, the CXCL1 concentration in brain tissue infected with the *ahr1*∆∆ mutant was similar to that infected with the WT strain (Fig. 3D); a similar pattern was observed for the related chemokine, CXCL2 (Fig. 3E). GM-CSF production is also induced by Ece1/CL in epithelial cells (14). Consistent with those data, the *ece1*∆∆ mutant induced less GM-CSF secretion than that observed in brains infected with WT; however, the *ahr1*∆∆ mutant induced GM-CSF secretion that was not statistically different from WT (Fig. 3E). The fungal burden established by the *ece1*∆∆ mutant is similar to the *ahr1*∆∆ mutant (see below) and higher than WT as previously demonstrated by Drummond (9) and Swidergall (15); therefore, the differences in cytokine/chemokine secretion between the two mutants are unlikely to be due to variation in fungal burden. These observations suggest that Ahr1 and Ece1 have both overlapping and distinct effects on cytokine/chemokine secretion and neutrophil recruitment to the brain during *C. albicans* infection.

### Ahr1 and Ece1 have distinct effects on brain CXCL1/2 and GM-CSF levels during *C. albicans* infection

**Fig 4:**
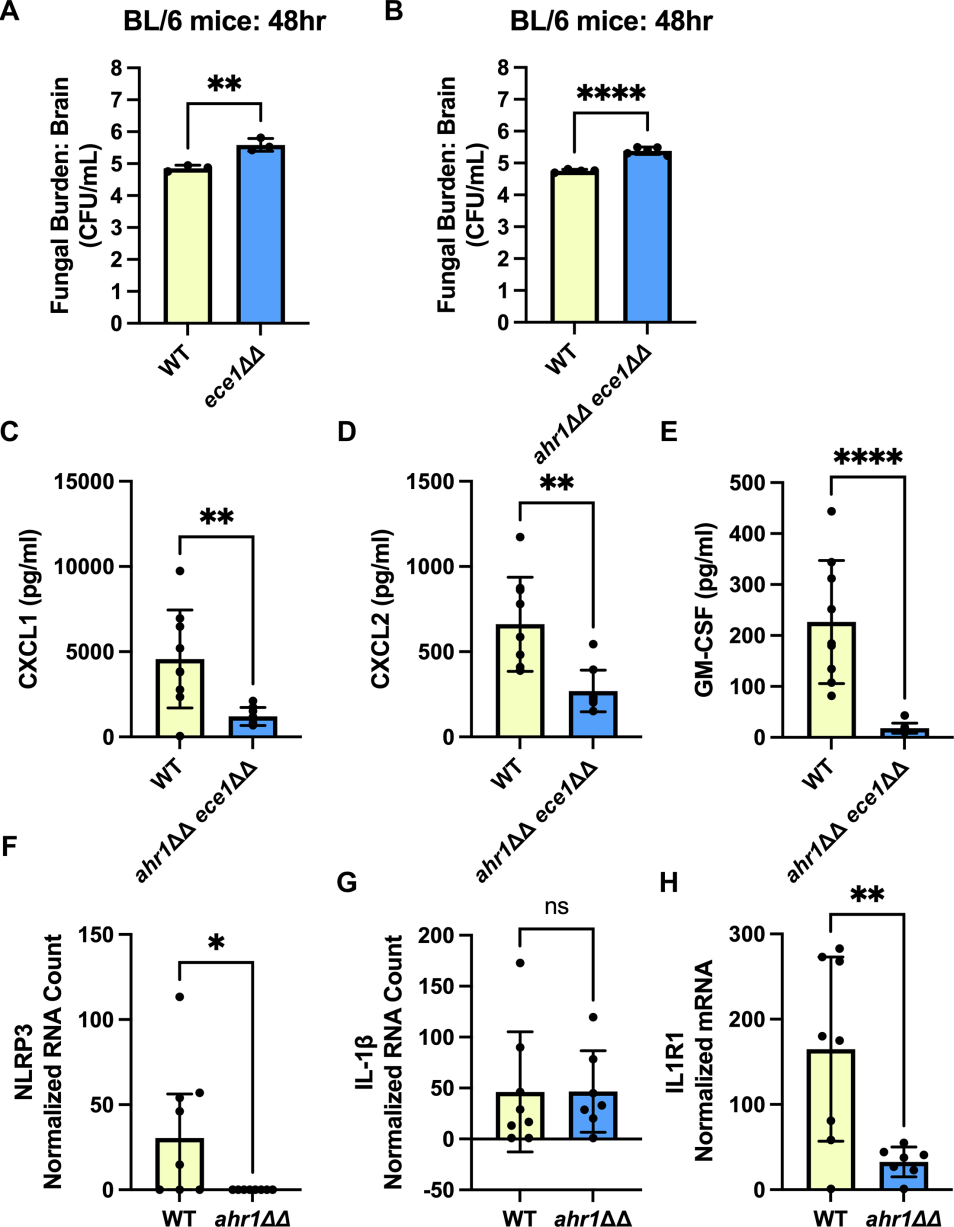
Expression of CXCL1/2 and GM-CSF in brains infected with the *ahr1*∆∆ mutant is Ece1/CL-dependent. (**A and B**) BL/6 mice (*n* = 5/group) were infected with WT, the *ece1*∆∆ mutant (**A**), or the *ahr1*∆∆ *ece1*∆∆ mutant (**B**) by tail-vein injection. Brain fungal burden was determined at 12 and 48 h post-infection. Statistical difference between groups was determined by two-tailed Student’s *t*-test on log-transformed fungal burden data (A: **P = 0.0048; B: **P = 0.0034; ****P = 0.0001). (**C–E**) BL/6 mice (*n* = 10/group) were infected with the indicated strains by tail-vein injection, and brains were harvested 24 h post-infection. The tissue was homogenized and assayed for cytokine/chemokine concentration as described in Materials and Methods. Differences between groups were analyzed by one-way ANOVA and Tukey’s multiple comparisons test. (**C**) CXCL1 concentrations: ***P = 0.0053. (**D**) CXCL2 concentrations: **P = 0.0013. (**E**) GM-CSF concentrations: ****P < 0.0001. (**F–H**) BL/6 mice (*n* = 7/group) were infected with WT *C. albicans,* and brains were harvested 24 h post-infection. Total RNA was extracted from the tissue and analyzed by NanoString nCounter to determine the expression of host NLRP3 (**F**), IL-1β (**G**), and IL1R (**H**). Difference between the two groups was analyzed by Welch’s unpaired *t*-test: NLRP3: *P = 0.024; IL-1β: NS: *P* > 0.05; IL1R: **P = 0.0072.

As discussed above, one of the reasons we tested the effect of Ahr1 on *C. albicans* brain infection was that it governs the expression of *ECE1* during filamentation *in vitro* (13). While Ece1/CL and Ahr1 have similar effects on fungal brain burden, neutrophil recruitment, and cytokine secretion, there are clear differences in CXCL1/2 and GM-CSF profiles. If Ahr1 is required for *ECE1* gene expression, Ece1 processing, or CL activation, then deletion of *ECE1* in the *ahr1*∆∆ mutant would not be expected to have a dramatic effect on phenotypes because the two genes would be epistatic to one another (16). Therefore, we generated an *ece1*∆∆ *ahr1*∆∆ double mutant and tested its effect on fungal burden and chemokine secretion. As previously reported (9, 15), the fungal burden of the brains of BL/6 mice infected with the *ece1*∆∆ single mutant was increased at 48 h relative to WT (Fig. 4A). Infection with the *ahr1*∆∆ *ece1*∆∆ double mutant also resulted in an increased fungal brain burden relative to WT (Fig. 4B). If Ahr1 and Ece1 were functioning independently, then the fungal burden of the *ahr1*∆∆ *ece1*∆∆ double mutant would be higher than either single mutant using the multiplicative model of genetic interactions (16). Predicted increase in fungal burden of the double mutant relative to WT = [(fungal burden of *ahr1*∆∆ mutant) − (fungal burden of WT)] + [(fungal burden of *ece1*∆∆ mutant) – (fungal burden of WT)]: 1.2 log10 CFU/mL + 0.7 log10 CFU/mL = 1.9 log10 CFU/mL predicted increase in fungal burden for the double mutant relative to WT. The observed brain fungal burden for the double mutant was 5.38 log10 CFU/mL, which is 0.6 log10 CFU/mL higher than WT, but less of an increase than is predicted if the two genes are functioning independently (Fig. 4B). The lower fungal burden for the double mutant indicates that there is a positive genetic interaction between *ECE1* and *AHR1* with respect to the fungal burden phenotype. This is consistent with Ahr1 and Ece1/CL functioning in the same pathway, suggesting that Ahr1-regulated genes may directly or indirectly Ece1/CL function or its downstream effects on the host.

Next, we asked if brain tissue levels of CXCL1, CXCL2, or GM-CSF in mice infected with the *ahr1*∆∆ mutant were Ece1/CL-dependent. Indeed, brain tissue infected with the *ahr1*∆∆ *ece1*∆∆ double mutant had dramatically reduced concentrations of CXCL1 (Fig. 4C), CXCL2 (Fig. 4D), and GM-CSF (Fig. 4E). The relative reduction in CXCL1, CXCL1, and GM-CSF brain tissue levels observed in mice infected with the *ahr1*∆∆ *ece1*∆∆ double mutants compared to WT-infected mice matched those observed for the *ece1*∆∆ mutant alone (Fig. S1), strongly supporting the hypothesis that Ece1/CL drives the induction of those cytokines in the *ahr1*∆∆ mutant and that Ece1/CL retains function in the *ahr1*∆∆ mutant.

NLRP3 inflammasome activation is a two-step process (17). A priming step is required to induce transcription of NLRP3 along with the gene encoding the pro-form of IL-1β. After transcription and translation, the NLRP3 inflammasome assembles and mediates the processing of substrates such as pro-IL-1β and the cytolytic pore gasdermin in a second step. Ece1/CL triggers the second step of NLRP3 inflammasome activation but has no effect on the priming step (18, 19). One explanation for the epistatic relationship between Ahr1 and Ece1 is that Ahr1 may play a role in the priming step. To test that hypothesis, we determined the transcript levels of NLRP3 and IL-1β in the brains infected with either WT or the *ahr1*∆∆ mutant 24 h post-infection. As shown in Fig. 4F, NLRP3 expression is significantly lower in brains infected with the *ahr1*∆∆ mutant compared to WT. In contrast, IL-1β expression is similar in the brains of mice infected with WT and *ahr1*∆∆ mutant strains (Fig. 4G). The expression of IL-1R is also reduced in the brains of mice infected with the *ahr1*∆∆ mutant compared to those infected with WT (Fig. 4H), although this likely reflects reduced IL-1β secretion because IL-1R expression is regulated by feedback activation (17). The reduced expression of NLRP3 in brains infected with the *ahr1*∆∆ mutant is consistent with our hypothesis that Ahr1 and Ece1 may affect different steps of the NLRP3 assembly and activation process.

### Ahr1 is not required for the expression of *ECE1* during brain infection

**Fig 5:**
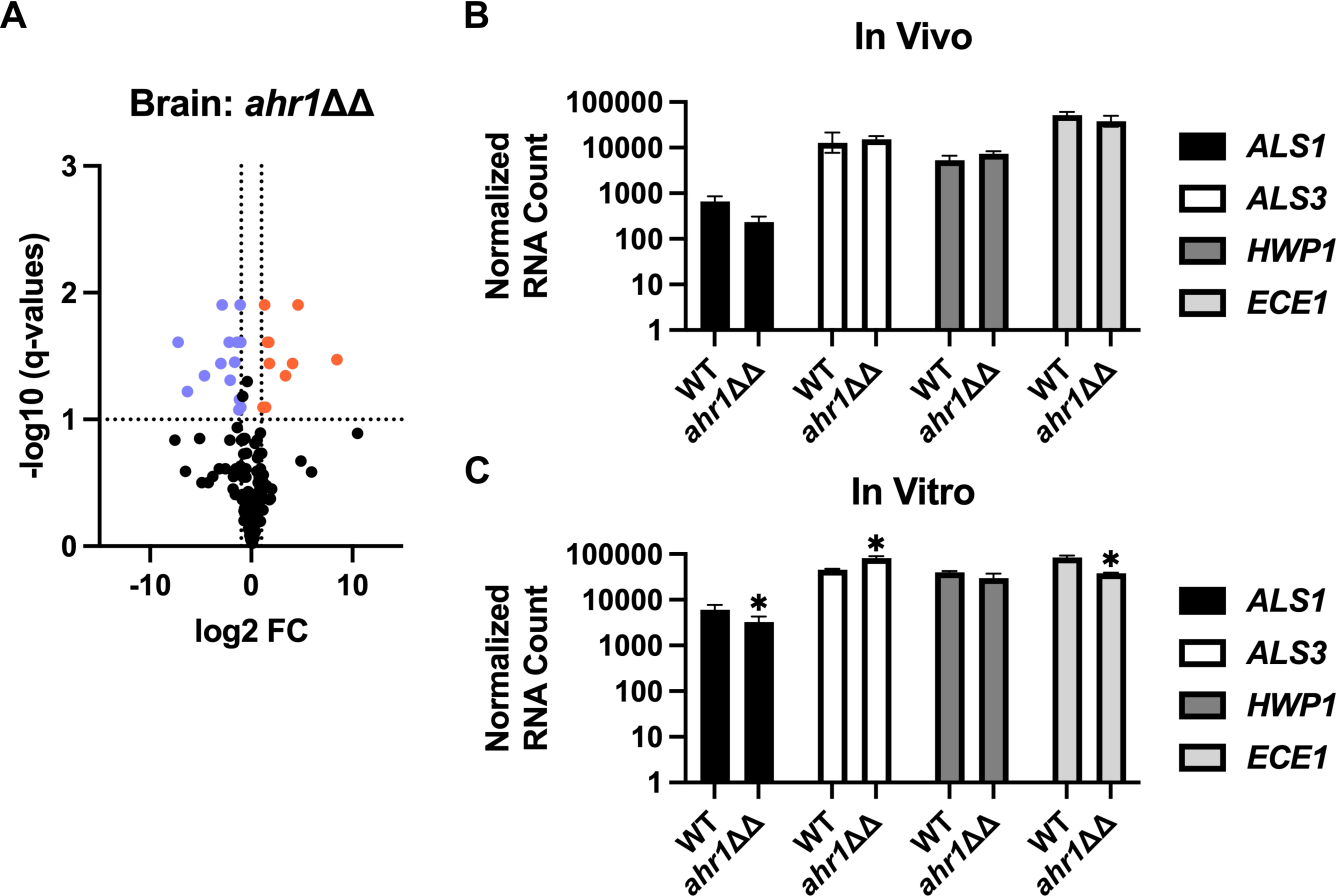
Ahr1 does not govern the expression of *ECE1* in *C. albicans*-infected brain tissue. *il1r*^−/−^ mice (*n* = 3–4/group) were infected with WT or the *ahr1*∆∆ mutant by tail-vein injection. Brain and kidney tissue were harvested, and total RNA was isolated as described in Materials and Methods. The mRNA counts for 185 *C. albicans* genes were measured using a NanoString nCounter. (**A**) Volcano plot shows differentially expressed genes (blue down; red up) as defined by ±2-fold change with FDR <0.1 (Benjamini-Hochberg). (**B**) Expression of hyphae-related genes in WT and *ahr1*∆∆ mutant during brain infection. None of the groups are statistically significantly different (one-way ANOVA using *P* = 0.05 as limit of significance). (**C**) Effect of Ahr1 on the expression of indicated genes relative to WT *in vitro* after 4 h of incubation in hyphae-expressing media (RPMI + 10% bovine calf serum). Difference in expression determined by Student’s *t*-test corrected with Benjamini-Hochberg (*P < 0.1).

Ruben et al. reported that *ECE1* expression was reduced in the *ahr1*∆∆ mutant *in vitro* with synthetic minimal medium supplemented with 10% serum (13). However, our results are not entirely consistent with the model that Ahr1 regulates *ECE1* expression. Specifically, one potential reason for the difference between the effect of the *ahr1*∆∆ and *ece1*∆∆ mutants on cytokine concentrations in the brain is that Ahr1 may not govern *ECE1* expression *in vivo* to the extent that it does *in vitro*. To test this hypothesis, we used NanoString nCounter technology to characterize the expression of 186 environmentally responsive *C. albicans* genes (including *ECE1*) in WT and *ahr1*∆∆ mutant strains in infected brain tissue (20). For this experiment, we used the *il1r*^−/−^ mice because the overall fungal burden 48 h post-infection was higher than that of the BL/6 mice and, therefore, provided a more robust level of fungal RNA. A volcano plot comparing the expression of WT and the *ahr1*∆∆ mutant in brain tissue is shown in Fig. 5A (See Table S1 for raw and processed data; significant differences in expression were defined as ±1 log2 FC, FDR < 0.1 using the Benjamini-Hochberg method).

In contrast to the *in vitro* experiment reported by Ruben et al. (13), there was no statistically significant difference in *ECE1* expression between the WT and the *ahr1*∆∆ mutant strains during brain infection (Fig. 5B). In addition, the expression of three other hyphae-associated genes (*ALS1*, *ALS3*, and *HWP1*), including two (*ALS3* and *HWP1*) Ruben et al. found to be dependent on Ahr1, was also not significantly different between the WT and *ahr1*∆∆ mutant strains (13). We also determined the expression profiles of the two strains in kidney tissue (Table S1); the expression of *ECE1* was slightly reduced in the *ahr1*∆∆ mutant (FC: 0.48; FDR = 0.0005). It is important to keep in mind that *ECE1* expression is essentially zero in yeast phase cells and is increased nearly 100,000× during infection and hyphae induction (20, 21). Therefore, it is unlikely that a 2-fold change in *ECE1* expression represents a biologically significant difference.

We had previously reported that the *C. albicans* gene in the expression in RPMI +10% serum correlated reasonably well with that observed in infected kidney (22). Therefore, we used the same NanoString nCounter to analyze the effect of the *ahr1*∆∆ mutation on gene expression after 4 h *in vitro* hyphal induction (Table S2). As shown in Fig. 5C, *ECE1* expression was reduced in the *ahr1*∆∆ mutant *in vitro* to an extent similar to that observed in kidney tissue (FC 0.43; FDR 0.001); *ALS1* expression was also modestly affected *in vitro,* while *ALS3* expression was increased and there was no effect on *HWP1*. Ruben et al. observed a ~10-fold reduction in *ALS3* and *ECE1* expression in the *ahr1*∆∆ mutant in their *in vitro* conditions (13). Thus, it seems that the specific environmental conditions play an important role in the genes that Ahr1 governs. Furthermore, the minimal effect of the *ahr1*∆∆ mutation on *ECE1* expression is consistent with the Ece1-dependent expression of CXCL1, CXCL2, and GM-CSF in the *ahr1*∆∆ mutant and further supports the notion that Ece1 retains significant function in the *ahr1*∆∆ mutant.

### *SAP5* promotes brain infection but does not play a role in Ahr1-mediated clearance of *C. albicans*

**Fig 6:**
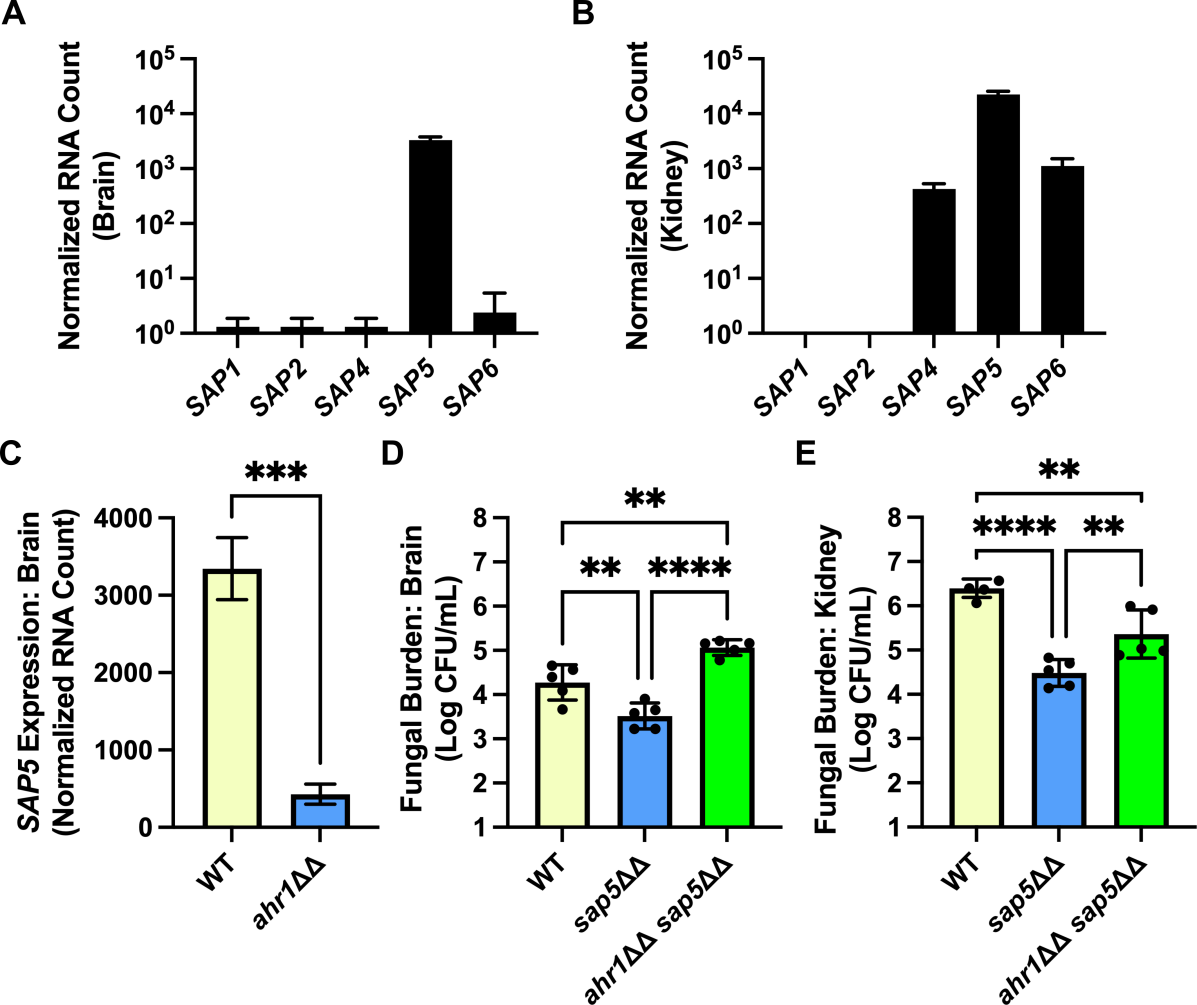
Ahr1 regulates *SAP5* expression in brain tissue, but it does not mediate the clearance of *C. albicans* from the brain. *In vivo* expression of SAP genes in the brain (**A**) and kidney (**B**). (**C**) Ahr1 is required for *SAP5* expression in *C. albicans*-infected brain (FDR *** <0.0001). BL/6 mice (*n* = 5/group) were infected with WT, the *sap5*∆∆ mutant, and the *ahr1*∆∆ *sap5*∆∆ mutant by tail-vein injection. Brain (**D**) and kidney (**E**) fungal burden was determined at 48 h post-infection. Statistical difference between groups was determined by one-way ANOVA with Tukey’s multiple comparisons test on log-transformed fungal burden data (D and E: **P < 0.001; ****P < 0.0001).

A striking difference between the expression profile for *C. albicans* in the brain compared to the kidney is that the secreted aspartyl protease *SAP5* is the only member of this multi-gene family that is expressed in brain tissue at 48 h (Fig. 6A), while *SAP4*, *SAP5*, and *SAP6* are all reasonably expressed in the kidney at the same time point (Fig. 6B). Furthermore, Ahr1 is required for *SAP5* expression in the brain (Fig. 6C) and kidney (Table S1). In mouse models of vulvovaginal candidiasis, *SAP5* deletion mutants induce lower IL-1β production and neutrophil recruitment (23). In addition, treatment of phagocytes with purified Sap proteins activates the NLRP3 inflammasome (24). Based on these observations, we wondered if Ahr1 governs expression of *SAP5,* which, in turn, is responsible for triggering IL-1-mediated clearance from the brain.

To test that hypothesis, we generated a *sap5*∆∆ single mutant and a *sap5*∆∆ *ahr1*∆∆ double mutant and compared the fungal brain burden for both strains to WT 48 h post-infection (Fig. 6D). Infection with the *sap5*∆∆ mutant led to reduced fungal brain burden (0.8 log10 CFU/mL) at 48 h. However, deletion of *AHR1* in the *sap5*∆∆ background suppressed this defect, and the double mutant retained the increased fungal brain burden of the *ahr1*∆∆ mutant. Mice infected with the *sap5*∆∆ mutant also had reduced fungal kidney burden, as did those infected with the *sap5*∆∆ *ahr1*∆∆ double mutant (Fig. 6E). These data reveal a previously underappreciated role for Sap5 during disseminated *C. albicans* infection.

If Ahr1-mediated *SAP5* expression activated the NLRP3 inflammasome, then the *sap5*∆∆ mutant would have phenocopied the *ahr1*∆∆ mutant and shown an increased fungal brain burden relative to WT. In addition, the double mutant should have shown a fungal burden similar to that observed for the *ahr1*∆∆ mutant. The brain fungal burden for the *sap5*∆∆ *ahr1*∆∆ double mutant was 0.8 log10 CFU/mL higher than WT, while that for the *ahr1*∆∆ mutant 1.2 log10 CFU/mL was higher than WT. By the multiplicative, genetic interaction model used above (16), the fungal burden for the *sap5*∆∆ *ahr1*∆∆ double mutant is predicted to be 0.4 log10 CFU/mL above WT if Sap5 and Ahr1 function independently (1.2 log10 CFU/mL + −0.8 log10 CFU/mL = 0.4 log10 CFU/mL). The observed brain fungal burden of the *sap5*∆∆ *ahr1*∆∆ double mutant is higher than predicted; the two mutants show a positive genetic interaction, which is consistent with Ahr1 regulating the expression of *SAP5*. However, other targets must be responsible for the role of Ahr1 in neutrophil recruitment and clearance of *C. albicans* from the brain.

## DISCUSSION

*C. albicans* CNS infection is not a common manifestation of invasive candidiasis and occurs in specific patient populations with the highest prevalence in premature infants and people with primary immune deficiencies involving the CARD9 innate immune signaling pathway (6–8). In mouse models of disseminated candidiasis, *C. albicans* initially infects the brains of immune-competent mouse strains, but the fungus is cleared by post-infection days 3–5, depending on inoculum and mouse/fungal strains (10, 25). CARD9/IL-1 signaling pathways contribute to the clearance of *C. albicans* from the brain through a process that is heavily dependent on the recruitment of neutrophils to the site of infection (7, 9). In this work, we have found that the transcription factor Ahr1 is required to activate IL-1 responses in the brain, which, in turn, recruit neutrophils and mediate the clearance of *C. albicans* from the CNS. Prior to this work, Ece1/CL has been demonstrated to mediate these *C. albicans*-induced neutrophil recruitment and fungal clearance in the brain (9, 25). As we will discuss below, we feel that the work reported here on the role of Ahr1 in *C. albicans* immune responses and fungal clearance from the brain has added to the seminal studies of Drummond et al. in multiple ways (7, 9).

We anticipated that Ahr1 would govern the expression of Ece1 as it has been reported *in vitro* (13) and, thereby, be required for the recruitment of neutrophils to the *C. albicans*-infected brain, leading to the clearance of the fungus. *In vivo* expression profiling, however, indicates that Ahr1 does not play an appreciable role in the expression of *ECE1* in the brain (Fig. 5B). In addition, *ece1*∆∆ and *ahr1*∆∆ mutants do not have identical effects on the cytokine/chemokine responses during brain infection, which would be expected if Ahr1 directly regulated *ECE1* expression (Fig. 3). This distinction is most clearly demonstrated by the differential effect of the *ece1*∆∆ and *ahr1*∆∆ mutants on the production of CXCL1, CXCL2, and GM-CSF in *C. albicans*-infected brain tissue (Fig. 3C and D). Specifically, the production of these three molecules is not affected by the loss of Ahr1 function but is highly dependent on Ece1. This result implies that Ece1 remains at least partially functional in the *ahr1*∆∆ mutant. Further supporting that conclusion, deletion of *ECE1* in the *ahr1*∆∆ mutant reduces CXCL1, CXCL2, and GM-CSF to levels that are nearly identical to those observed in the brain tissue of mice infected with the *ece1*∆∆ single mutant (Fig. 4C through E; Fig. S2). Together, these data indicate that Ece1/CL induces CXCL1, CXCL2, and GM-CSF production through a mechanism that is Ahr1-independent.

At the same time, both Ahr1 and Ece1/CL are required for *C. albicans* to induce the production of IL-1β, IL-6, and, to a lesser extent, IL-1α in brain tissue. Furthermore, the *ahr1*∆∆ and *ece1*∆∆ show a positive genetic interaction analysis with respect to the clearance of *C. albicans* from the brain. This result is consistent with Ahr1 and Ece1 functioning in a linear pathway, which is the result to be expected if Ahr1 positively regulates the expression of *ECE1*. How, then, might this, at least, partially contradictory relationship between Ahr1 and Ece1 be reconciled? While the following model is consistent with our data and that reported by others, we fully recognize that (i) it is more a hypothesis, or working model, than a definitive mechanism; (ii) it is genetics-based, and thus, it is of modest resolution; and (iii) additional immunological studies with higher resolution will be required in the future.

Our data indicate that Ahr1 appears to have a more specific effect on the NLRP3 pathway, while Ece1/CL affects additional NLRP3-independent processes. This is supported by the almost undetectable levels of IL-1β in brain tissue infected with the *ahr1*∆∆ mutant, while IL-1α levels are much less affected than with the *ece1*∆∆ mutant (Fig. 3A and B). IL-1α is an alarmin, and while the inflammasome can be involved in its production, inflammasome-independent production/secretion is generally more common (14). Ece1/CL causes direct host membrane disruption, which is a well-characterized trigger of IL-1α secretion. We propose that Ahr1 is primarily required for activation of the NLRP3 inflammasome, which, in turn, drives IL-1β secretion and a modest level of IL-1α secretion. Ece1/CL, on the other hand, activates the NLRP3 inflammasome and causes direct cell lysis, leading to a robust production of both IL-1β and IL-1α.

IL-1α plays an important role in the production of CXCL1 in the infected brain. Therefore, we propose that the different effects of Ahr1 and Ece1/CL on CXCL1 and GM-CSF production are because sufficient IL-1α is produced in the *ahr1*∆∆ mutant to drive CXCL1 and GM-CSF production. Recent work reported by Hanaoka and Domae (14) has shown IL-1α is directly released from epithelial cells after exposure to Ece1/CL and, using neutralizing antibodies to IL-1α and IL-1β, demonstrated that IL-1α but not IL-1β is required to drive GM-CSF production. Taken together, these data and literature observations provide strong support for our model and for the conclusion that Ece1/CL expression, activation, and function of Ece1/CL are preserved in the *ahr1*∆∆ mutant during brain infection.

However, if CXCL1 production is maintained in the *ahr1*∆∆ mutant, then what explains the reduced neutrophil recruitment to the brain? Drummond et al. reported that CXCL1 was a key driver of neutrophil recruitment to the *C. albicans*-infected brain and that IL-1β contributed to its production (9). While the data that Drummond et al. reported clearly support the importance of the IL-1β-CXCL1 pathway in neutrophil recruitment to the brain (9), the effects of IL-1β and CXCL1 on this process do not have the same magnitude. Specifically, neutrophil recruitment in the brains of *C. albicans*-infected *il1b*^−/−^ mice was reduced by 8-fold compared to WT mice, while that in the brains of *cxcl1*^−/−^ mice was reduced by ~2-fold (9). These data, therefore, indicate that IL-1β has both CXCL1-dependent and -independent effects on neutrophil recruitment, with the CXCL1-independent pathway making a 4-fold larger contribution. Our data suggest that Ahr1 is primarily a driver of NLRP3-IL-1β responses, while Ece1/CL affects that process, as well as IL-1α-dependent responses.

Since both Ahr1 and Ece1/CL activate the NLRP3 inflammasome, how can their functions be differentiated? We propose that Ahr1 is required for the priming step of NLRP3 (17), while Ece1/CL is involved in the activation step. The priming step induces the transcription of NLRP3, which, after translation to its gene product, is assembled into the inflammasome and activated in the second step. Brain tissue infected with the *ahr1*∆∆ mutant has essentially no NLRP3 expression, strongly supporting this model. Ece1/CL, on the other hand, has been previously shown to drive the second activation step, but not the priming step of NLRP3 inflammasome activation (18, 19). This model explains how the *ahr1*∆∆ mutant can express and activate Ece1/CL, yet not trigger inflammasome-mediated IL-1β release; in short, there is little or no inflammasome in the *ahr1*∆∆ mutant for Ece1 to activate in the second step.

Ece1/CL must still cause some level of cell lysis in the *ahr1*∆∆ mutant and, thereby, trigger the production of substantial amounts of IL-1α, leading to CXCL1 and GM-CSF production as discussed above. The function of Ahr1 and Ece1/CL at different steps of inflammasome assembly and activation is also consistent with the positive genetic interaction observed with the *ece1*∆∆ *ahr1*∆∆ mutant; specifically, Ahr1 is required for the first step of the two-step NLRP3 activation pathway, while Ece1 is required for the second step. If either step is disrupted, then the outcome is similar, and thus, the two proteins show epistasis with respect to inflammasome activation (16).

While this model explains much of our data, there are alternatives. With that in mind, it is important to acknowledge an important limitation of this study: the cytokine/chemokine responses were measured at the whole organ level. The multiple cell types within the brain all participate in the innate immune response to CNS infection and include microglia, neurons, astrocytes, and endothelial cells. Therefore, it is possible and/or likely that Ahr1 interacts with different brain cell types compared, for instance, to Ece1/CL. As such, the distinctions between the effects of the *ahr1*∆∆ and *ece1*∆∆ mutants may be due to differences in cell type interactions. Indeed, a detailed examination of how brain cell types contribute to protecting the brain from *C. albicans* will no doubt yield fascinating new information.

In addition to the host response questions that remain, a key fungus-associated question is, which Ahr1 target or targets are responsible for inducing NLRP3 priming and activation? To our knowledge, Ece/CL and the SAPs are the only two *C. albicans* proteins that have been clearly linked to the inflammasome. Pattern recognition receptors such as the TLRs and TNF drive NF-κB-mediated expression of NLRP3 as part of the priming step (17). TLR2, TLR4, and TLR6, for example, are mediators of the host response to *C. albicans,* and all three recognize mannoproteins in the cell wall (26), suggesting that cell wall protein expression may be altered in the *ahr1*∆∆ mutant. This hypothesis would be consistent with previous *in vitro* expression profiling of Ahr1, but direct unbiased profiling during infection would likely be needed to identify candidate targets. In total, 1,3-β-glucan has also been shown to induce NLRP3 expression in a dectin-1-dependent manner, suggesting that Ahr1 may be required for dectin-1 interactions with the 1,3-β-glucan component of the *C. albicans* cell wall (27). Future studies to identify the targets of Ahr1 responsible for these phenotypes are likely to be challenging but potentially fruitful.

Finally, as part of our investigation of Sap5 as an Ahr1-dependent mediator of *C. albicans* clearance from the brain, we discovered that the *sap5*∆∆ mutant has significant fitness defects in infectivity in both the kidney and brain (Fig. 6D and E). The reduced fitness of the *sap5*∆∆ mutant in brain is consistent with our finding that *SAP5* is by far the predominant SAP expressed in *C. albicans*-infected brain tissue (Fig. 6A). In *C. albicans*-infected kidney, *SAP5* is expressed >10-fold higher than *SAP4* or *SAP6,* but the other two are expressed at much higher levels than observed in infected brain tissue (Fig. 6B); the kidney expression pattern for *SAP4/5/6* is consistent with that previously reported by Solis et al. in *C. albicans*-infected kidney (21). Although the role of the SAPs, particularly *SAP4/5/6*, in *C. albicans* pathogenicity has been found to be modest based on previous work (28), *SAP5* has demonstrated the most pronounced effect as a single mutant and was similar to the *sap4*∆∆ *sap5*∆∆ *sap6*∆∆ triple mutant. Our data also suggest that a contributing factor to the dominant infection phenotype of *SAP5* in the kidney is also likely due to its high level of expression relative to *SAP4* and *SAP6*.

In summary, we have found that Ahr1 is a transcription factor that governs the ability of *C. albicans* to trigger the NLRP3/IL1 immune response in the brain and, thereby, affects neutrophil recruitment as well as the clearance of the fungus from the brain. The role of Ahr1 in this process has both shared and distinct features compared to the Ece1/candidalysin. While many mechanistic details remain to be understood, our data are consistent with the conclusion that Ahr1 is required to prime the NLRP3 inflammasome assembly process. In contrast, Ece1/candidalysin affects the second, NLRP3 activation step while also triggering cell lysis-associated, NLRP3-independent host responses that contribute to neutrophil recruitment to the *C. albicans*-infected brain.

## MATERIALS AND METHODS

### Microbiological methods and strains

The reference and transcription factor mutant strains were from the library generated by Homann et al. (29) in the SN background and were obtained from the Fungal Genetics Stock Center. All *C. albicans* strains were pre-cultured overnight in yeast peptone dextrose (YPD) medium at 30°C, and standard recipes were used to prepare the YPD plates/media (29).

### Mammalian cell culture methods

hCMEC/d3 cells (Sigma-Aldrich, Cat# SCC066) were routinely grown in collagen-coated T75 non-treated cell culture flasks (Thermo, Cat# 156800) in growth medium: EBM-2 Basal Medium (Lonza, Cat# CC-3156) supplemented with 1 mL bovine brain extract (Lonza, Cat# CC-4092) with heparin, 250 µL hegf (Lonza, Cat# CC-4017C), 1.4 µM hydrocortisone (Sigma, Cat# H0888), 1% penicillin-streptomycin, and 5% fetal bovine serum (FBS; Cat# A52094-01) per 250 mL. Cells were incubated at 37°C in a humidified incubator with 5% CO_2_, and growth media were refreshed every 2 days. Cells were passaged using TrypLE Express (Gibco, Cat# 12604021) at ~80% confluency and were not used beyond passage 30. Differentiation media: 1% FBS, 1% penicillin-streptomycin, 1.4 µM hydrocortisone, 5 µg/mL ascorbic acid (Millipore Sigma Aldrich, Cat# A92902), 1% chemically defined lipid concentrate (Gibco, Cat# 12563011), and 10 mM HEPES (Gibco, Cat# 15630106).

### Strain construction

Standard lithium acetate transformation methods were used to construct additional *C. albicans* mutant strains from the SN152 or SN250 parental strain using the transient CRISPR/Cas9 method (30). The oligonucleotide and plasmid used to construct the mutant strains in this study are listed in Table S4. To generate independent isolates of the *ahr1*∆∆ mutant strain, both alleles of the *AHR1* gene were deleted in an SN250 background strain (31) with an *ARG4* cassette (32) which was amplified from the pFA-LAL plasmid using the AHR1.P1 and AHR1.P2 primer pair using sgRNA targeting both alleles of the *AHR1* gene. To generate the *ece1*∆∆ mutant, both alleles of *ECE1* were deleted with a *LEU2* cassette, which was amplified from the pSN-40 plasmid using the ECE1.P1 and ECE1.P2 primer pair using sgRNA targeting two alleles of the *ECE1* gene. The SN250 background was used to generate the *sap5*∆∆ mutant. To do this, both copies of the *SAP5* alleles were deleted by amplifying the *NAT1* cassette from the pNAT1 plasmid using primer pair SAP5.1 and SAP5.2 and using two sgRNAs targeting both alleles of the *SAP5* gene. To generate the *bcr1*∆∆ *ahr1*∆∆ double mutant, both copies of *AHR1* were deleted in the *bcr1*∆∆ mutant background using the *ARG4* cassette generated from the pFA-LAL plasmid using primer pair AHR.P1 and AHR1.P2. The same strategy was used to generate *ahr1*∆∆ *ece1*∆∆ double homozygous mutant. In this case, both copies of the *ECE1* gene were deleted in the *ahr1*∆∆ mutant background with the *ARG4* cassette, which was amplified from the pFA-LAL plasmid using primer pair ECE1.P3 and ECE1.P4 and using sgRNA targeting two alleles of the *ECE1* gene. To generate the *ahr1*∆∆ *sap5*∆∆ double mutant, both copies of *SAP5* were deleted in the *ahr1*∆∆ mutant background. To do this, the pNAT1 plasmid was used to amplify the *NAT1* cassette using SAP5.P1 and SAP5.P2 primer pairs and using sgRNA targeting two alleles of the *SAP5* gene. The resultant transformants were selected on SD plates lacking either leucine or arginine or on YPD plates supplemented with nourseothricin (NAT) (200 µg/mL). The correct integration of the deletion cassette was confirmed by PCR. None of the *AHR1*, *SAP5*, or *ECE1* mutants showed growth defects on standard YPD medium at 30°C (Fig. S4A and B).

### Mouse model of disseminated candidiasis

BL/6 female mice (five per group, 6–8 weeks old; Jax) or IL1RKO mice were inoculated by lateral tail-vein injection with 5 × 10^5^ CFUs of the indicated strains. Mice were monitored daily, and those who demonstrated symptoms of severe disease (fur ruffling, abnormal posture, and/or failure to respond to the surroundings) were euthanized immediately. Organs (brain and kidney) were harvested at 12, 24, or 48 h post-infection as described above. A serial dilution of lysates (10 µL of 10-fold dilution series) was spotted on YPD plates, and the quantitative fungal burden (CFU/mL) was determined. Statistical significance was determined on the log-transformed values using an unpaired *t*-test on one-way ANOVA, followed by correction for multiple comparisons when more than two groups were used.

### RNA isolation from *C. albicans*-infected organs

At 48 h post-infection, mice were euthanized following the protocol approved by the University of Iowa IACUC. Organs (brain and kidney) were harvested and collected into ice-cold RNA later solution. The brain/kidney was then transferred to a mortar and flash-frozen with liquid nitrogen. Using a pestle, the frozen organ was ground to a fine powder. The resulting powder was collected in a 5 mL Eppendorf tube, and 1 mL of ice-cold TRIzol was added. The samples were placed on a rocker at RT for 15 min, transferred into a 1.5 mL Eppendorf tube, and centrifuged at 10K RCF at 4°C for 10 min. The cleared TRIzol was collected into a 1.5 mL Eppendorf tube, and 200 µL of RNase-free chloroform was added to each sample. The tubes were shaken vigorously for 15 s, kept at RT for 5 min, and then centrifuged at 12K rpm at 4°C for 15 min. The cleared aqueous layer was collected into a new 1.5 mL Eppendorf tube, and RNA was further extracted following the Qiagen RNeasy Kit protocol (cat# 74134, Qiagen).

### Expression profiling using NanoString nCounter

NanoString-based expression profiling was performed as described previously (23). Briefly, total RNA isolated from *in vivo* samples was used for hybridization with a custom NanoString probe set (Tables S1 and S2) or a probe set for mouse stock NanoString probe set of inflammation-related genes including NLRP3, IL-1β, and ILR1. For kidney samples, 80 ng of total RNA was used per reaction, whereas 3.5 µg of total RNA was used for brain samples. RNA and probe set mixes were hybridized at 65°C for 18 h according to the manufacturer’s protocol. Following the hybridization reaction, samples were processed to the nCounter prep station, and samples were scanned on an nCounter digital analyzer. nCounter .RCC files for each sample were imported into nSolver software to evaluate the quality control metrics. Background subtraction was performed using negative control probes to establish a background threshold, which was then subtracted from the raw counts. The resulting background-subtracted total raw RNA counts underwent a two-step normalization process. First normalized against the highest total counts from the biological triplicates and then to the wild-type samples. The statistical significance of changes in gene expression was determined using the Benjamini-Hochberg method with a cutoff FDR of 0.1 and a ±2-fold change in expression.

### Measurement of brain concentrations of cytokines, chemokines, and myeloperoxidase (MPO) activity

For the quantification analysis of cytokine and chemokine protein concentrations in the whole brain, mice were infected intravenously with *C. albicans* with WT, *ece1*∆∆ mutant, *ahr1*∆∆ mutant, or *ece1*∆∆ *ahr1*∆∆ double mutant. Twenty-four hours post-infection, the brains were harvested, weighed, and homogenized in PBS and a protease inhibitor cocktail (Sigma). The organ homogenates were clarified by centrifugation, aliquoted, and stored at −80°C until use. The supernatants were clarified, and concentrations of IL-1α, IL-1β, CXCL1, and CXCL2 were quantified using a Luminex Magnetic bead array (R&D Systems). MPO levels were measured by enzyme immunoassay (Hycult Biotech) following the manufacturer’s protocol.

### Isolation of immune cells from *C. albicans*-infected brain tissue

Briefly, 5 × 10^5^ CFUs were injected intravenously into female BL6 mice (two per group, 6–7 weeks old, Jax). At 24 h post-infection, mice were anesthetized, and brains were harvested following trans-cardiac perfusion with ice-cold PBS to remove the circulating blood cells. The brains were collected into a Petri dish and minced into small pieces using sterile razor blades and transferred to 15 mL conical tubes containing digestion buffer composed of HBSS with calcium and magnesium (Gibco) supplemented with collagenase D (500 mg; Roche) and DNase I (100 mg; Roche). Samples were incubated for 30 min at 37°C with gentle rocking, then collected in six-well plates and triturated with a plunger and collected into 15 mL tubes. Specifically, 2.5 mL of ice-cold RP10 buffer (RPMI 1640; Gibco) supplemented with 12% heat-inactivated fetal bovine serum (FBS; Gibco) and 1% penicillin-streptomycin was added, and cells were pelleted at 310 × *g* for 5 min at 4°C. The supernatant was removed carefully, and the pellets were resuspended in 3 mL of HBSS + solution (HBSS without Ca and Mg + 2 mM EDTA + 0.5% BSA). The cells were gently triturated three times and filtered sequentially through 100 µm and 70 µm cell strainers to obtain a single-cell suspension. The cells were pelleted at 310 × *g* for 5 min at 4°C and resuspended in 6 mL of 20% room temperature Percoll. The samples were centrifuged at 310 × *g* for 20 min at 4°C with low deceleration. The myelin layer and supernatant were carefully aspirated, and the cells were resuspended in HBSS+.

### Flow cytometric quantification of brain neutrophils

Following Percoll cleanup, cells were resuspended in 500 µL of HBSS+ and split into a round-bottom, 96-well plate (250 µL/well). Cells were pelleted at 310 × *g* for 5 min at 4°C with low deceleration set at 8 and incubated with purified rat anti-mouse CD16/CD32 (BD Biosciences) to block the Fc receptor for 10 min on ice. Cells were incubated for 30 min at 4°C in the dark with the following antibodies: PE-conjugated anti-mouse CD11b (eBioscience), FITC-conjugated anti-mouse CD45 (eBioscience), and PerCP-eFluor 710-conjugated anti-mouse Ly-6G (eBioscience). Fluorescence minus one controls were included to accurately set neutrophil gates during flow cytometry. After antibody incubation, cells were pelleted and washed with HBSS+ and resuspended in 250 µL HBSS+ with LIVE/DEAD Fixable Orange (602) dye (Invitrogen) per manufacturer’s instructions (1:1,000). Cells were incubated for 30 min at 4°C in the dark, and cell fluorescence was then acquired on an Attune NxT flow cytometer with a CytKick autosampler. Data were analyzed in Attune NxT software version 6.2.3269.1.

### Software

GraphPad Prism (V. 10.6.0) was used to plot data and perform the statistical tests.

## SUPPLEMENTAL MATERIAL

Supplemental Figures mbio.01492-26-s0001.pdf Figures S1 to S4.

Table S1 mbio.01492-26-s0002.xlsx *In vivo* expression profile of the *ahr1*∆∆ mutant.

Table S2 mbio.01492-26-s0003.xlsx *In vitro* profile of the *ahr1*∆∆ mutant.

Table S3 mbio.01492-26-s0004.docx Strains used in this work.

Table S4 mbio.01492-26-s0005.xlsx Oligonucleotides used in this work.

ASM does not own the copyrights to Supplemental Material that may be linked to, or accessed through, an article. The authors have granted ASM a non-exclusive, world-wide license to publish the Supplemental Material files. Please contact the corresponding author directly for reuse.
