# Supplementary material for: The transcription factor Ahr1 governs the recruitment of neutrophils to the brain and clearance of Candida albicans during CNS infection
Source: mBio. 2026 Jul 20;17(8):e01492-26. doi: 10.1128/mbio.01492-26 (PMC13463782; doi:10.1128/mbio.01492-26)
Supplement: Supplementary file 1 [file mbio.01492-26-s0001.pdf]

Fig. S1

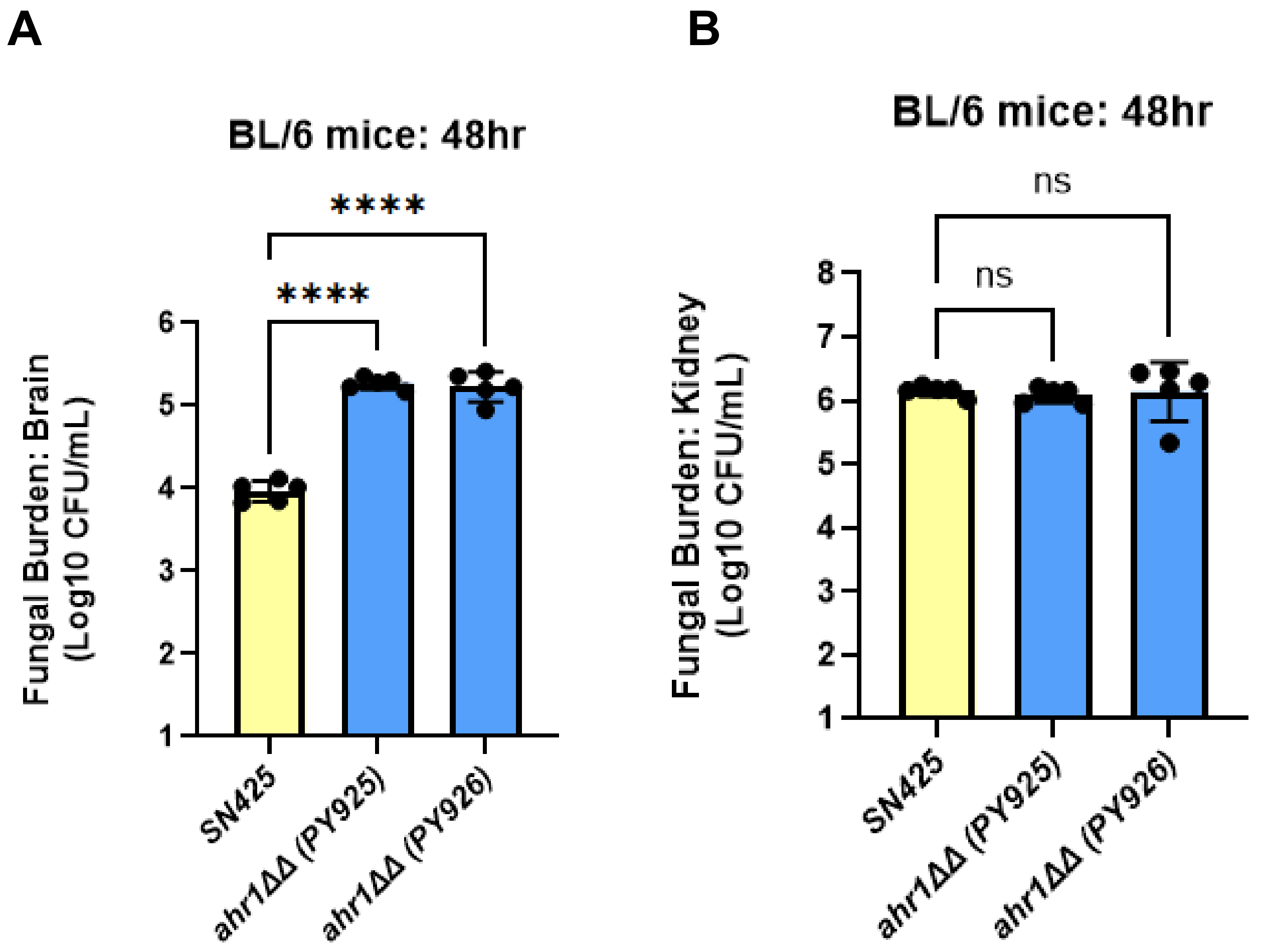

Figure S1. Multiple independent isolates of Ahr1 have same in vivo phenotypes. BL/6 mice (n = 5/group) were infected with *C. albicans* WT or two independent isolates of the *ahr1*ΔΔ mutant by tail-vein injection and brain (**A**) or kidney (**B**) fungal burden was determined at 48hr post-infection. Statistical differences between groups were determined One-way ANOVA using Tukey's correction for multiple comparisons. **** P<0.0001; NS P >0.005).

Fig. S2

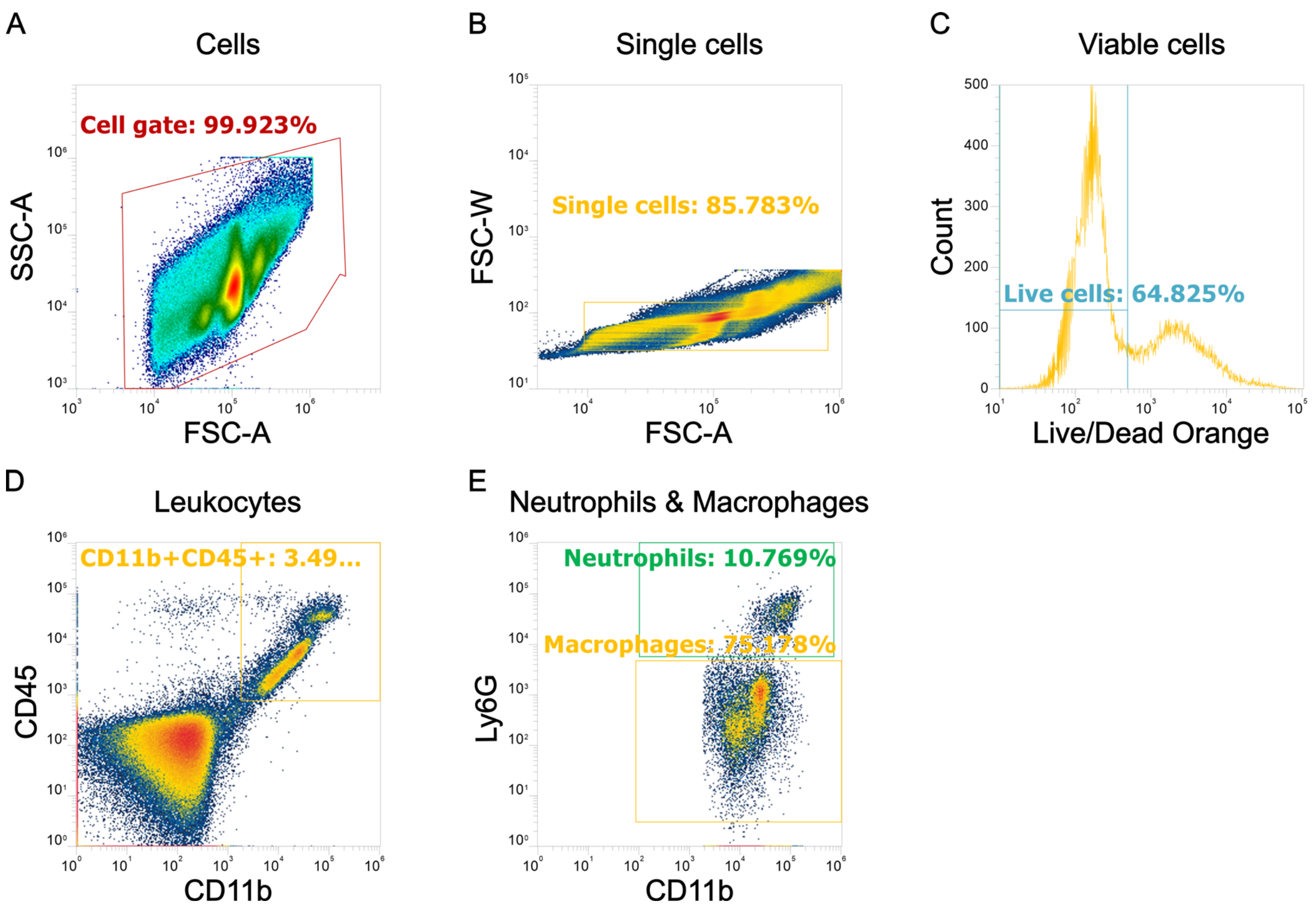

Figure S2. Flow cytometry gating strategy for neutrophils. A. Debris was eliminated by gating on intact cells. **B**. Within the cell gate, single cells were isolated by excluding cells with higher FSC width, indicative of clumped cells. **C**. Within the single cell gate, inviable cells were identified by high live/dead stain and not included in the viable cell gate. **D.** Within the viable cells gate, CD45 high and CD11b high cells were isolated. **E**. Within the CD45+CD11b+ gate, Ly6G high cells were identified as neutrophils. The final gated population was CD45+CD11b+Ly6G+(high).

Fig. S3

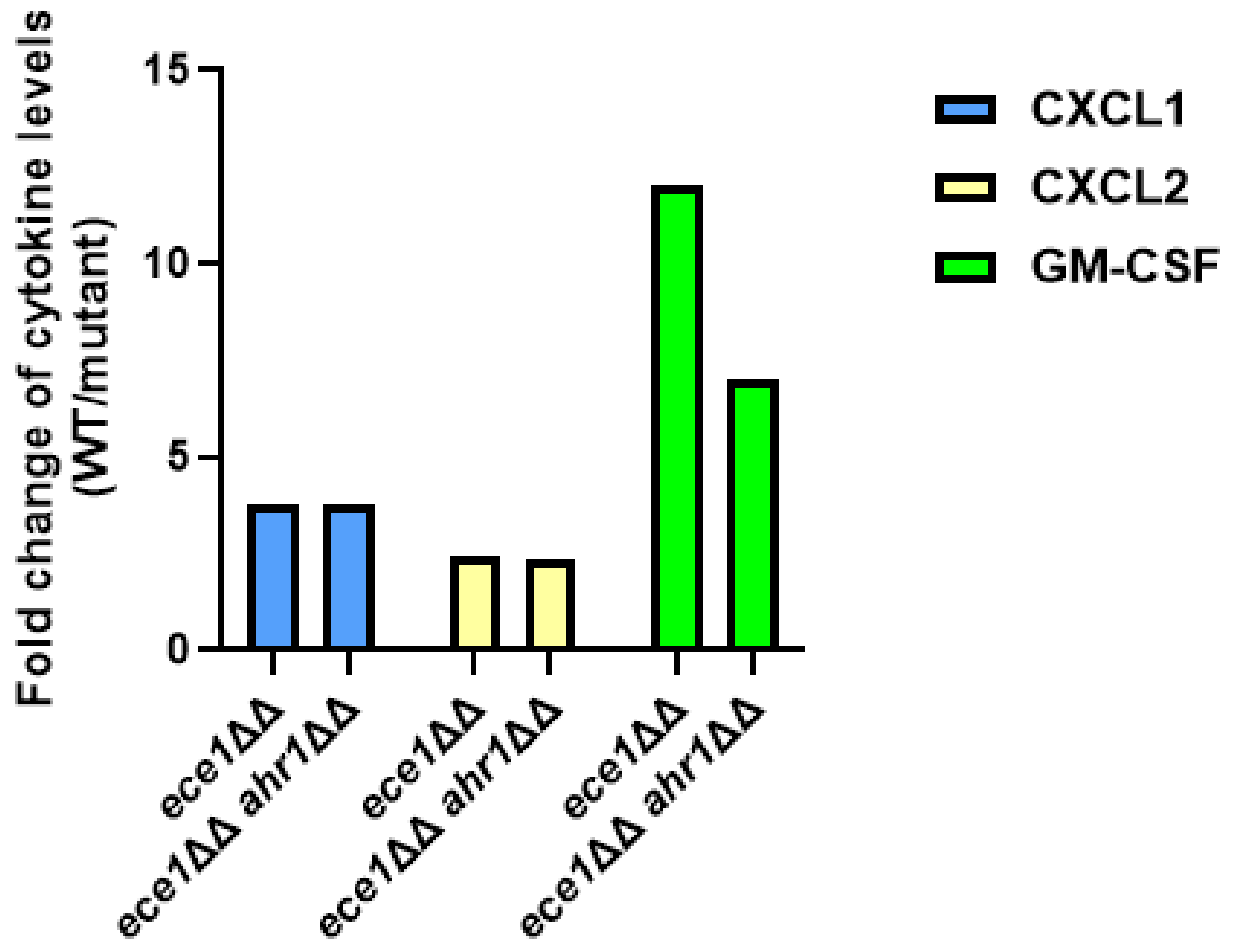

Figure S3. The effect of the *ece1*ΔΔ mutant on CXCL1/2 and GM-CSF is similar to the *ece1*ΔΔ *ahr1*ΔΔ mutant. The fold change in chemokine production from **Fig. 3&4** was determined by normalizing the effect of the indicated mutants to WT and expressed as WT/mutant to provide a positive number.

Fig. S4

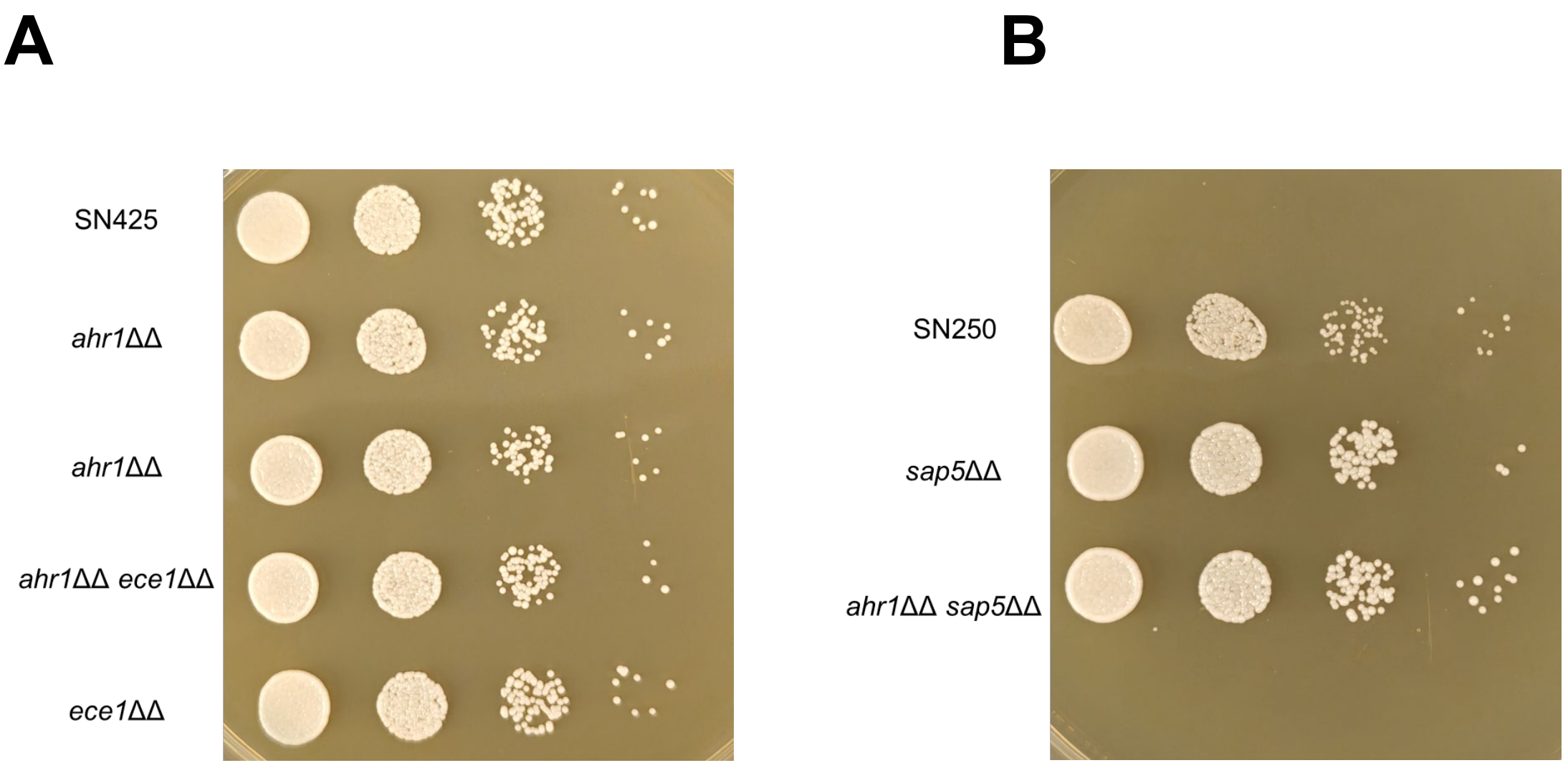

Figure S4. Deletion of *AHR1*, *SAP5*, or *ECE1*, either singly or in combination, does not affect growth on YPD at 30ºC. **A&B**. The indicated strains were grown overnight in YPD, diluted to 1 OD_{600}. This culture along with further 10X, 100X, and 1000X dilutions were plated on YPD agar and incubated for 24hr at 30ºC. The plates were photographed at that time point.
