# Supplementary material for: The transcription factor Ahr1 governs the recruitment of neutrophils to the brain and clearance of Candida albicans during CNS infection
Source: mBio. 2026 Jul 20;17(8):e01492-26. doi: 10.1128/mbio.01492-26 (PMC13463782; doi:10.1128/mbio.01492-26)
Supplement: Supplementary file 2 [file mbio.01492-26-s0004.docx]

**Table S3. Strain Table**

| **Strain** | **Genotype** | **Reference** |
| --- | --- | --- |
| SN152 | CAI4 with *iro1::IRO1/iro1*::*λ*imm434 *his*1::*hisG*/*his1*::*hisG leu2∆/leu2∆ arg4∆/arg4∆* | 31 |
| SN250 | *arg4*Δ/*arg42*Δ *URA3*/*ura3*Δ::*imm 434* *IRO1/iro1Δ*::*imm 434* | 31 |
| SN425 | *ura3Δ::λimm434::URA3-IRO1/ura3Δ::λimm434 arg4::hisG::CdARG4/arg4::hisG his1::hisG/his1::hisG leu2::hisG::CdHIS1/leu2::hisG::CmLEU2* | 32 |
| *ahr1Δ/Δ* | As SN152 with *ahr1Δ:: HIS1/ ahr1Δ:: LEU2* | 29 |
| *ahr1Δ/Δ* | As SN250 with *ahr1Δ :: ARG4/* *ahr1Δ :: ARH4* | This Study |
| *ece1Δ/Δ* | As SN152 with *ece1Δ :: LEU2/* *ece1Δ ::LEU2* | This Study |
| *sap5Δ/Δ* | As SN250 with *sap5Δ:: NAT1/ sap5Δ:: NAT1* | This Study |
| *ahr1Δ/Δ*  *ece1Δ/Δ* | As *ahr1Δ/Δ* with *ece1Δ :: ARG4/ece1Δ :: ARG4* | This Study |
| *ahr1Δ/Δ*  *sap5Δ/Δ* | As *ahr1Δ/Δ* with sap*5Δ :: NAT1/sap5Δ :: NAT1* | This Study |
